# Women’s knowledge and associated factors in preconception care in adet, west gojjam, northwest Ethiopia: a community based cross sectional study

**DOI:** 10.1186/s12978-017-0279-4

**Published:** 2017-01-25

**Authors:** Yitayal Ayalew, Amlaku Mulat, Mulugeta Dile, Amare Simegn

**Affiliations:** 1 Department of midwifery, College of Health Sciences, University of Debre Tabor, Debre Tabor, Ethiopia; 2 Department of Midwifery, College of Health Sciences Mekelle University, Mekelle, Ethiopia

**Keywords:** Preconception care, Awareness, Reproductive age group women, Adet, Ethiopia

## Abstract

**Background:**

Preconception care is the provision of biomedical, behavioural and social health interventions to women and couples before the occurrence of conception to improve their health status. There is poor maternal and child health and lack of knowledge in developing countries about preconception care. Therefore, this study aimed to assess women’s knowledge and associated factors in preconception care in Adet Town, Gojjam, Northwestern Ethiopia.

**Methods:**

A community based cross-sectional study was conducted among 422 systematically selected reproductive age group women who are living in the Adet town from March 1 to 30, 2016. The data were collected using pre tested and structured questionnaires through face-to-face interviews. The data were entered into Epi-Info version 3.5, and cleaned and analysed using SPSS version 20. Descriptive summary of the data and logistic regression were used to identify possible predictors using odds ratio with 95% confidence interval and *P*-value of 0.05.

**Results:**

The study revealed that the overall knowledge of preconception care was 27.5% (95% CI: 23.2, 32.0). Women who attended secondary educational and whose age is from 25 to 34 years were more likely to have better knowledge on preconception care than their counterparts were; (AOR 6.52, CI 2.55, 16.69) and (AOR 4.10, CI 1.78, 9.44) respectively. However, Women who had no history of family planning use were 85% less knowledgeable than those who had a history of family planning use (AOR: 0.15; 95% CI: 0.05, 0.44).

**Conclusions:**

In this finding level of women’s knowledge of preconception care is relatively low. Having a history of family planning use, having high levels of educational status, and being older age were associated with good knowledge. This finding suggests that there is a need to give emphasis and deliver health education about preconception care for women in order to increase their knowledge.

## Plain english summary

According to World Health Organization, preconception health care is an essential component of reproductive health which focuses on the conditions and risk factors that could affect a woman if she became pregnant.

In this study, respondents were asked via close ended and structured questionnaire face to face interviews whether or not they know preconception health care services.

Of the total 422 respondents, more than half of participants 251 (59.5%) were married and 113 (26.8%) of women were housewives.

The finding revealed that only 27.5% of of the respondents had knowledge of preconception care.

The possible determinants identified were: being educated, having a history of contraceptive use, and age.

In conclusion, women’s knowledge of preconception care is relatively low In Adet Town. This finding suggests that there is a need to give emphasis and deliver health education about preconception care for women in order to increase their knowledge.

## Background

Preconception care (PCC) is the provision of biomedical, behavioural and social health interventions to women and couples before the occurrence of conception and aims at improving their health status, and reducing behaviours and individual and environmental factors that contribute to poor maternal and child health outcomes [1]. It is imperative that preconception care is seen as an earlier opportunity, not just for family planning or to reduce maternal and neonatal mortality, but also to improve long-term outcomes for adolescent girls, women, and children. Adolescents’ general health and reproductive health must increasingly be considered as crucial stages in the continuum of care. Preconception care is widely recognized as a way to optimize women’s health through biomedical and behavioural changes prior to conception, ultimately to improve pregnancy outcomes [2]. In terms of prevention, PCC is primary prevention for the future baby and secondary prevention for prospective mothers [3].

Preconception care is important to reduce several risk behaviours and exposures that can affect fetal development and subsequent outcomes [4]. Therefore, it should be planned to address reproductive system problems, to reduce environmental hazards, toxins and medications that are known teratogens, to promote nutrition and folic acid intake, to advise on weight management, to detect problems related to genetic conditions, family history, substance use, chronic diseases and infectious diseases, to advise on vaccinations, family planning, psychosocial concern, domestic violence, and housing.

Despite the interventions in place, progress in maternal and child health outcomes over the last 20 years has been slow globally. Studies showed that less than 1/3^rd^ of women of childbearing age visited the health institutions and speak with a health care provider prior to pregnancy about their health status and its potential impact on pregnancy outcome [5].

As of 2011 sub-Saharan Africa report on maternal health shows that in sub-Saharan Africa there is a poor preconception care practice and this is due to low economic status, lack of health care providers, being illiterate and poor awareness about maternal health including preconception care [6]. Preconception care (PCC) is the most important maternal health care service to reduce maternal and child mortality and morbidity by identifying and treating any risks early, promoting health, and preventing disease. In addition to this, PCC highly associated with increase antenatal care, delivery care and post natal care service utilizations which are the corner store to improve maternal and child health [7].

Different articles show that the women’s preconception care knowledge and practice in developing countries including Africa is low. Studies conducted in Sri Lanka, Nigeria and Sudan showed that the women’s level of knowledge regarding with preconception care is very low [8–11].

Worldwide, there are different literatures done in order to assess factors associated with women’s knowledge in preconception care and they revealed that women’s knowledge regarding to preconception care was affected by women’s age, ethnicity, occupation, educational status, pregnancy intention, previous history of abortion, monthly income, previous history of stillbirth, parity and family planning [12–14].

Preconception care is a neglected but a critical component of maternal and child health care services [15]. Therefore, in settings where there is low awareness of preconception care, promotion of preconception care among reproductive age group women is important to boost maternal health care services and to reduce complications during antenatal care, institutional delivery and post natal care. However, family planning, utilization, and factors associated with it have not been well understood in resource limiting settings like Ethiopia.

Therefore, the main aim of this study was to describe the level of women’s awareness regarding to preconception care which will help in estimating the preconception care needs of reproductive age group women and which in turn could help to prepare the necessary resources and flourish programs for better reproductive health services. The other main purpose of the current study was addressing the knowledge gap with regard to factors associated with awareness in preconception care among reproductive age group women. Understanding the factors benefit in a way that women as well as care givers intervene on those factors. This study is also believed to benefit many concerned stakeholders in decision making and policy development.

## Methods

### Setting

**Fig. 1:**
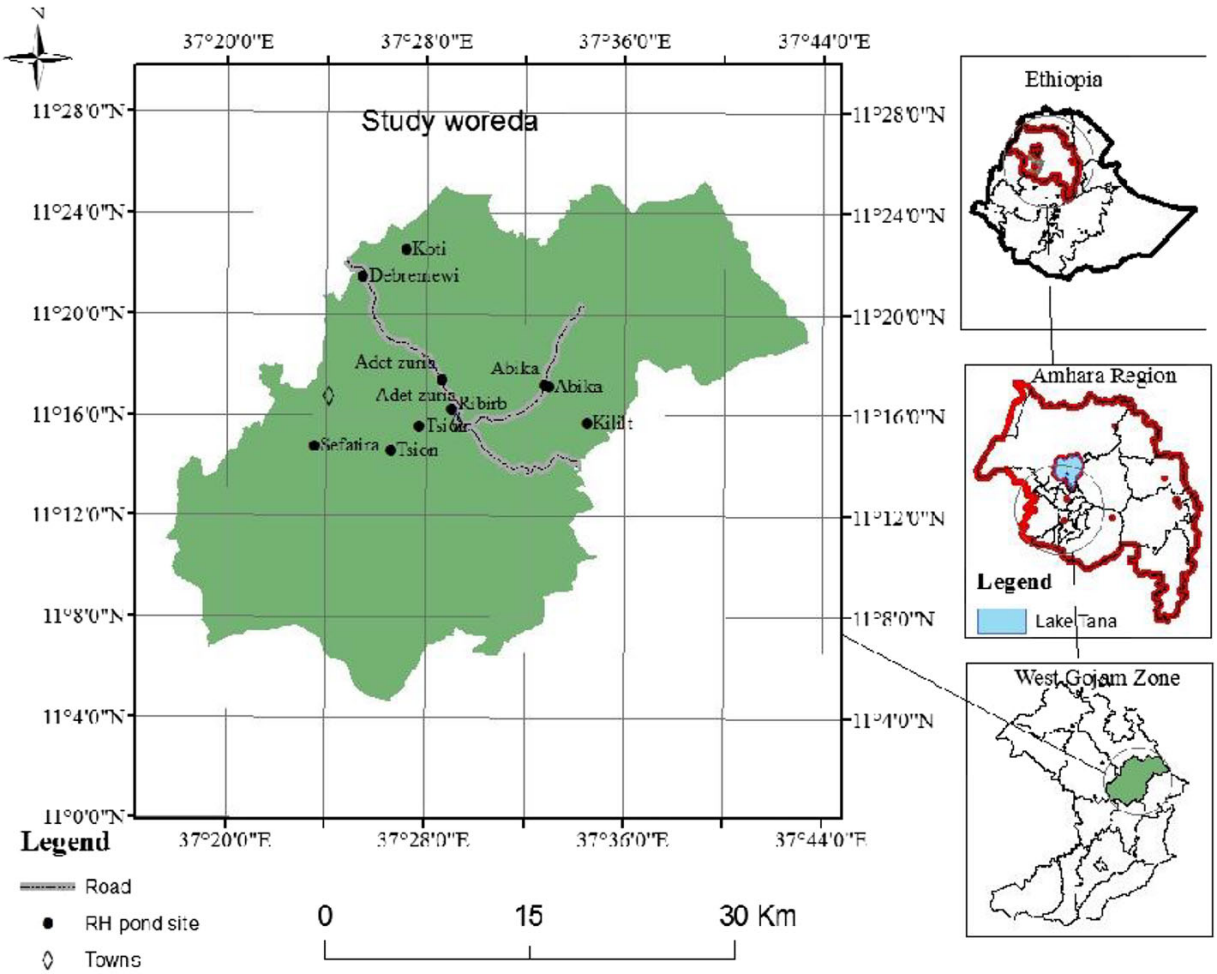
Location of Adet town

This Community based cross sectional study was conducted in Adet town from March 01–30, 2016. Adet Town is an administrative town of Yilmana Densa Wereda and located in West Gojjam Zone, Amhara Regional State. It is located 524 km away from Addis Ababa and 42 km far from Bahirdar. It has an altitude of 2, 216 m above the sea level (Fig. 1).

According to the figures from the Central Statistical Agency in 2015, the estimated total population of the town was 42, 983. Out of this 21, 234 (49.4%) were men and 21, 749 (50.6%) were women. The total number of women who were in reproductive age group (15–49 years) were 14, 248 which accounts 33.1% of the total population [16].

### Participants

All women who live in Adet were the source population and all reproductive age group women who live in Adet were the study population. finally, individual reproductive age group women were the stud units. All reproductive age group women who have lived in Adet for 6 months and above were included.

### Sampling technique and procedure

A sample size of 422 was determined using single population proportion formula. *n* = Z^2^ p (p −1)/d^2^ with the following assumptions: proportion (P) of a population practicing family planning to be 50% since there was no study conducted in Ethiopia, a confidence level (CI) of 95%, and marginal error (d), 5% and 10% non-response rate.

All the three Kebeles (the smallest unit of the district) of Adet were taken. To reach the study unit systematic sampling technique was used in the Kebeles. The first house was selected randomly in one place and every 32^nd^ house for all kebele was asked. The sampling interval of the households in each Kebele was determined by dividing the total number of households in the specific kebele to the allocated sample size. When there was no a reproductive age group woman in the selected house, nearby house was asked. In case of more than one eligible woman were encountered in the selected household, a lottery method was used to determine which woman would be interviewed.

### Variables

The dependent variable was the Knowledge on preconception care, and the independent variables were demographic, socioeconomic characteristics, obstetric history, social behaviour, women’s health status and associated factors.

### Operational definition

#### Preconception care

Any interventions either advice or treatment, and lifestyle modification before being pregnant.

#### Knowledge

Level of women’s knowledge on preconception care was measured based on correct response using fifteen preconception care knowledge questions and the question was scored out of 34 points. With 50% of cut of point women’s knowledge was divide into two.

#### Good knowledge

Those who have scored 17–34 of correct responses to preconception care knowledge questions.

#### Poor knowledge

Those who have scored less than 1–16 correct responses to preconception care knowledge questions.

#### Smoking status

Had a history of smoking or currently smoke regardless of amount.

#### Alcohol consumption

Consumption of alcoholic drinks on other than holidays and culturally special ceremony days.

### Data collection

Data was collected by three diploma nurse interviewers using a pretested structured questionnaire at working hours’. The questionnaire was translated into local language, Amharic by experts in both languages and was translated back to English by another person to ensure consistency and accuracy. The data collection process was closely supervised by two health officers and the principal investigator. The data collectors and supervisors were recruited based on previous experience on data collection and fluency in the local language. In addition, training was given for two consecutive days on how to interview, handling ethical issues and maintaining confidentiality and privacy. The pre-test study covered 21 reproductive age group women who are living in merawi woreda, which become out of the main study two weeks before the commencement of the main data collection.

Pre-test was conducted to familiarize enumerators with the administration of the interview process and for ensuring consistency. Debriefing sessions were held with the pre-test field staff and the questionnaires were modified based on lessons drawn from the pre-test. Completed questionnaire crosschecked daily for inconsistencies and completeness.

### Data analysis

Data was first checked manually for completeness and then coded, entered and cleaned by EPI-Info 3.5.3 statistical software. Then the data were exported to SPSS windows version 20 for data checking, cleaning and logistic regression. Cleaning was done by calculating frequencies and sorting. Bivariate analysis between dependent and independent variables was performed using binary logistic regression. *P* <0.25 was used as criteria to select candidate variables for multivariate analysis. Multivariable logistic regression analysis was done to adjust for possible confounding variables. *P*-value <0.05 with 95% confidence interval (CI) for OR (odds ratio) was used in judging the significance of the associations. Results were presented in text, tables and charts.

### Ethical consideration

Ethical clearance and approval was obtained from the Ethical review committee of the College of Medicine and Health Science, Mekele University. In addition, the official letter of cooperation granted by the administrative offices of Adet Town. The purpose of the study was explained to the study participants and written informed consent was secured before data collection was started and confidentiality of the information was ensured by coding. Participation was on a voluntary basis after written consent, and responses were kept confidential. The consent procedure was approved by the ethics committee for all. The interview was undertaken privately in separate area.

## Results

### Socio demographic characteristics

A total of 422 subjects were participated with a response rate of 100%. The median age of the participants was 25 years with an inter quartile range of 11 years. Four hundred twenty one (99.8%) of the participants were Amhara and 358 (84.8%) were Orthodox Christian. Hundred and fourty nine (35.3%) of respondents had a monthly household income of less than or equal to 70 Dollar and 131(31%) were literate. More than half of participants 251 (59.5%) were married and 113(26.8%) of women were housewives. One hundred and twelve (25.4%) and 114(19.8%) of the participant’s husband were market trade vendor and primary school respectively (Table 1).

**Table 1:** Socio-demographic characteristic of women in Adet, Gojjam, Northwestern Ethiopia, 2016 (*n* = 422)

Characteristics	Frequency (*N*)	Percent (%)
Age		
15-24	196	46.4
25-34	136	32.2
35-49	90	21.3
Ethnicity		
Amhara	421	99.8
Shinasha	1	.2
Religion		
Orthodox	358	84.8
Muslim	60	14.2
Protestant	4	1.0
Occupation		
Housewife	113	26.8
Market trade vendor	107	25.4
Civil servant	79	18.7
Student	95	22.5
Daily laborer	28	6.6
Educational status		
No formal education	99	23.5
Primary school	97	23.0
Secondary school	131	31.0
College and above	95	22.5
Monthly income		
≤ 70 Dollar	149	35.3
70.5-117.5 Dollar	96	22.7
118-177.5 Dollar	55	13.1
178-250 Dollar	65	15.4
> 250	57	13.5
Marital status		
Married	251	59.5
Divorced	35	8.3
Widowed	17	4.0
Single	119	28.2
Husband’s educational status (*n* = 251)		
No formal education	47	18.7
Primary school	112	44.6
Secondary school	54	21.5
College and above	38	15.2
Husband’s occupation (*n* = 251)		
Market trade vendor	114	45.4
Governmental employee	84	33.5
Daily laborer	37	14.7
Farmer	16	6.4
Communication		
Mobile	287	68.0
Television	199	47.2
Radio	92	21.8
None	65	15.4

NB. For communication the total summation of percentage is more than 100% due to multiple answers

**Table 2:** Women’s knowledge on general concept of preconception care in Adet, West Gojjam, North Western, Ethiopia, 2016 (*n* = 422)

Variables	Frequency (*N*)	Percentage (%)
Ever heard		
Yes	134	31.8
No	288	68.2
Preconception care needed		
For men, only	6	1.4
For women, only	18	4.3
For men and women	60	14.2
Don’t know	338	80.1
Preconception care important		
For baby, only	22	5.2
For mother, only	39	9.2
For baby and mother	70	16.6
Don’t know	291	69.0
Site for preconception care		
Home	5	1.2
Health institution	73	17.3
Home and health institution	56	13.3
Don’t know	288	68.2
Total	422	100

### Obstetrics characteristics

**Fig. 2:**
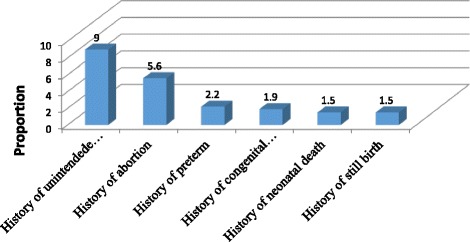
Obstetrics characteristics of women in Adet, West Gojjam, Northwestern Ethiopia, 2016 (*n* = 422)

Two hundred sixty eight (63.5%) has been pregnant before. Of the 268, 168 (62.7%) of them had ≤ 2 alive children and 100 (37.3%) of them had 3 or more alive children. One hundred and fifty four (36.4%), 53(12.6%), 191 (45.3%) and 24 (5.7%) of participants were nulliparous, primi para, multipara and grand multiparous respectively. The majority (58.1%) of respondents had a history of family planning use (Fig. 2). From 5 women who had history of congenital anomaly baby, two of them were counseled for subsequent pregnancy.

### Health status and social behaviour of women

**Fig. 3:**
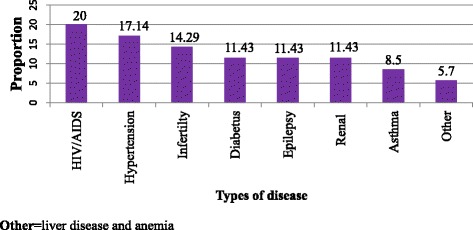
Types of chronic health problem women have in Adet, west Gojjam, North Western Ethiopia, 2016 (*n* = 35)

Thirty-five (8.3%) of respondents had a chronic health problem and from those who had chronic health problem 7 (20%) of them were known HIV positive women (Fig. 3). Four hundred twenty one (99.8%) of participants were not cigarette smoker and only 1 woman (0.2%) was addicted to alcohol.

### Preconception care knowledge score

Among the total of 422 participants, 134 (31.8%) of women have heard about preconception care before. For those who have heard about preconception care; the major source of information was health institution 69 (51.5%) and minority 9 (6.7%) of them have heard from friends. Fourty (29.8%), 32 (23.9%) and 14 (10.5%) of them have heard from the mass media, school and family/relatives respectively (Table 2).

The minimum and maximum score of participants was 1 and 34 respectively. One hundred and sixteen (27.5%) (95% CI: 23.2, 32.0) of them had good knowledge on preconception care (Table 3).

**Table 3:** Women’s knowledge on preconception health and behavioural risk factors on fetus in Adet, West Gojjam, North Western, Ethiopia, 2016 (*n* = 422)

Variables	Frequency (*N*)	Percentage (%)
Diabetes mellitus		
Yes	88	20.9
No	334	79.1
Epilepsy		
Yes	83	19.7
No	339	81.3
Obesity		
Yes	87	20.6
No	335	79.4
STIs and HIV/AIDS		
Yes	138	30.3
No	284	69.7
Heart disease, including hypertension		
Yes	87	20.6
No	335	79.4
Stress and depression		
Yes	78	18.5
No	344	81.5
Genetic problem		
Yes	55	13.0
No	367	87.0
Cigarette smoking		
Yes	141	33.4
No	281	66.6
Alcohol consumption		
Yes	143	33.9
No	279	66.1
Exposure to environmental hazard		
Yes	72	17.1
No	350	82.9
Illegal drug intake		
Yes	99	23.5
No	323	76.5
Gender based violence, including FGM		
Yes	66	15.6
No	356	84.4

**Table 4:** Women’s knowledge on preconception care component in Adet, West Gojjam, Northwestern Ethiopia, 2016 (*n* = 422)

Variables	Frequency(*N*)	Percentage (%)
Take folic acid		
Yes	66	15.6
No	356	84.4
Weight should be maintained		
Yes	123	29.1
No	299	70.9
Modify diet		
Yes	113	26.9
No	309	73.1
Regular exercise		
Yes	80	19.9
No	342	80.1
Substance should be avoided		
Yes	133	31.5
No	289	68.5
Avoid cigarette smoking		
Yes	127	30.1
No	295	69.9
Avoid alcohol consumption		
Yes	119	28.2
No	303	71.8
Avoid illicit drugs		
Yes	101	23.9
No	321	76.1
Create healthy environment		
Yes	123	29.1
No	299	70.9
Free from environmental radiation		
Yes	103	24.4
No	319	75.6
Free from environmental chemical		
Yes	100	23.7
No	322	76.3
Free from stressors		
Yes	106	25.1
No	316	74.9
Total	422	100.

### Knowledge on preconception health and behavioural risk factors on fetus

Regarding women’s knowledge on preconception health and behavioural risk factors; alcohol consumption (33.9%), cigarette smoking (33.4%) and STIs including HIV/AIDS are most frequently mentioned issues, whereas gender based violence (15.6%), and genetic problem (13%), were the least frequently mentioned issues (Table 5).

**Table 5:** Association between different variables and women’s level of knowledge on preconception care in Adet, West Gojjam, North western Ethiopia, 2016(*n* = 422)

Variables	Level of knowledge		COR (95%CI)	AOR (95%CI)
	Poor (%)	Good (%)		
Age				
15–24	165(84.2)	31(15.8)	1	1
25–34	86(63.2)	50(36.8)	3.10(1.84 -5.20)**	2.38 (1.14-4.95) **
35–49	55(61.1)	35(38.9)	3.39(1.91- 6.00)**	4.10 (1.78-9.44) **
Educational status				
No formal education	86(86.9)	13(13.1)	1	1
Primary school	72(73.4)	25(25.8)	2.30(1.10-4.81)**	3.37 (1.35-8.42) **
Secondary school	95(72.5)	36(27.5)	2.51 (1.25-5.04)**	3.85 (1.54-9.56) **
College and above	53(55.8)	42(44.2)	5.24 (2.58-10.63)**	6.52 (2.55-16.69) **
History of family planning use				
Yes	155(63.3)	90(46.7)	1	1
No	151(85.3)	26(14.7)	0.30(0.18-0.48)**	0.15(0.045-0.44) **

NB: **indicates *p*-value <0.05, CI = confidence Interval

### Knowledge on changes should be made before pregnancy

Based on women’s knowledge on what things should be made before pregnancy: pregnancy should be planned were mentioned by 100% of women, visit health institution (31.8%), and screened and treated for disease (31.8%) before pregnancy were the most frequently listed, whereas get a vaccination (14.6%) and family palming use (11.8%) were the least frequently listed issues (Table 4).

### Predictors of knowledge for preconception care

Results of binary logistic regression showed that Women whose age is more than 25 years, who are civil servants, who attended more than primary education, whose monthly income is between 118 and 177.5 Dollar, whose husband attended college and above, whose husband is civil servant, were identified as significant predictors of knowledge for preconception care while Women who had no history of family planning use were not associated.

In multivariable logistic regression three variables, i.e., women’s age, history of family planning and educational status were associated. When compared with those who are not able to write and read, women who attended more primary education were more than three times more likely to know preconception care (AOR: 3.37; 95% CI: 1.35, 8.42). In addition, women whose age is above 25 years were 2.38 times more likely to know preconception care than their counterparts (AOR: 2.38; 95% CI: 1.14, 4.95).

However, women who had no history of family planning use were 85% less likely to know preconception care than women who had a history of family planning use (AOR: 0.15; 95% CI: 0.05, 0.44) (Table 5).

## Discussion

The study revealed that overall knowledge of preconception care by reproductive age group women was 27.5%, which was higher than studies carried out in Sudan (11.1%) [11], Iran (10.4%) and Nepal (15.6%) [17, 18]. The highest knowledge in this study might be due to the fact that there were varied in time and large and representative sample size, which make the study comprehensive. However, it is significantly lower than the findings from Saudi Arabia (37.9%), Jordan (85%) [19, 20], Egyptian (76%) and Arabian (51%) mothers [21]. The low knowledge level in this study might be due to the relative low media coverage in Ethiopia, which showed there is a need to broaden media coverage in the country. In addition, it is lower than a study conducted in the USA among low income Mexican American group (76%) [12]. This might be due to the fact that the study population in the USA was women who presented in women’s care clinic and most of the participants had higher levels of educational status. Whereas, this study was community based and most women were not educated. It may be also due to the absence of strategies and policies of the Ethiopian Ministry of Health regarding addressing Preconception care.

In the study, measure factors that influenced knowledge of preconception care were education, age, and history of family planning use.

It was observed that women who attended primary education were more than three times more knowledgeable than women who had no formal education (AOR: 3.37; 95% CI: 1.35, 8.42). The finding of this study is not consistent with studies conducted in Nigeria and Sudan [11, 22]. This might be due to the difference in study population and large sample size in our study. Similarly, women who had secondary school education were four times more likely knowledgeable than women who had no formal education (AOR: 3.85; 95% CI: 1.54, 9.56). The finding of this study is consistent with a study done in Iran [23], Nigeria and Sudan [11, 22]. This might be due to the fact that when the women’s educational level is increasing, there might be exposed to information regarding to preconception care. The more educated women may be motivated to know about health and risk factors and they might have interest to read, listen and watch any information sources.

In addition, women who attended college and above education were nearly seven times more likely had better knowledge than those who had no formal education (AOR: 6.52; 95% CI: 2.55, 16.69), which was in line with the findings reported from the Netherlands, USA and Nigeria [24–26], Iran, Sri Lanka, and USA, where the women’s level of awareness of preconception care was increased with their educational status [8, 12, 17]. However, this finding is not consistent with studies done in Jordan, Nepal and Ghana [18, 20, 27]. This might be due to the fact that education improves communication with partner, women’s status in the community and the influence of education on women’s decision making skill to search source of information.

Similarly, women whose age is from 25 to 34 years were more than two times more knowledgeable than those whose age is from 15 to 24 years (AOR: 2.38; 95% CI: 1.14, 4.95). This is in line with a finding from USA [24]. This might be explained by women who are in these age groups are mostly at high risk to be pregnant and give birth and seek information about preconception health and social behaviour risk factors and other things related to preconception care.

On the other hand, women who were in the age group of 35–49 years were 4 times more likely had better knowledge about preconception care than those who were in the age group of 15–24 years (AOR: 4.10; 95% CI: 1.78, 9.44). The result of this study is consistent with studies conducted in the Netherlands, Italy, Nigeria [26, 28, 29], China, Iran and USA [12, 17, 30]. This might be due to the fact that women’s knowledge on preconception care was increased with their age. However; this finding is not consistent with studies conducted in Jordan, Nepal [18, 20], Nigeria, Ghana and Sudan [11, 22, 27]. This might be due to the fact that our study was community based, conducted in large sample size and the study population were rural women who relatively have no access of information, which showed that there is a need to settle a strategy to address this issue.

Finally, this study noted that women who had no history of family planning use were 85% less knowledgeable than those who had a history of family planning use (AOR: 0.15; 95% CI: 0.05,0.44). This finding is in line with studies conducted in China, France, and Sudan as all of the studies showed that use of contraception was positively associated with women’s knowledge in preconception care [11, 31, 32]. This might be due to the fact that pregnancy counselling, including preconception care is being given in the family planning unit, women who used family planning might have information regarding preconception care. However, the finding of this study is not consistent with a study conducted in Francisco general hospital [33]. This might be due to relative large sample size and community based study design of our study, which makes our study sounding. In addition, this might be due to the access of information in the Francisco general hospital about their contraceptive use, but Ethiopian women may need to visit health institutions as it was the major source of information.

However, this study does have some inherent limitations. First, the study design makes it difficult to determine the direction of causality and there is a risk of social desirability bias and interviewer bias. In addition, this study was not triangulated which might be difficult to get new factors, and suggested to be studied in the future. Finally, though there are wide ranges of factors which affect knowledge of preconception care among reproductive age group women, only individual level factors were addressed in this study. Hence, considering factors from the service providers’ side and structural barriers would have been important.

## Conclusions

The finding of this study showed that women’s knowledge on preconception care is low. This finding revealed that only a woman’s age, educational status, and history of family planning use were statically associated with women’s knowledge in preconception care. It indicated that being elder, having a high educational level, and having a history of family planning use were increased women’s level of knowledge of preconception care. Therefore, establishing preconception care strategies which can address all the components of the care and advocating women’s education and family planning use are important.

## Abbreviations

AOR: Adjusted odds ratio
HIV: Human immune deficiency virus
NTDs: Neural tube defects
PCC: Preconception care
USA: United states of america
